# Accuracy of declared nutrient content on labels of commercial complementary food products in Cambodia, Indonesia and Senegal

**DOI:** 10.1111/mcn.13504

**Published:** 2023-03-24

**Authors:** Mary Champeny, Katelyn Yuen‐Esco, Eva Juniza, Ndeye Y. Sy, Rosenette Kane, Jane Badham, Anzélle Mulder, Alissa M. Pries

**Affiliations:** ^1^ Helen Keller International New York New York USA; ^2^ JB Consultancy Johannesburg Gauteng South Africa

**Keywords:** nutrition labelling

## Abstract

Commercially produced complementary foods (CPCF) have the potential to fill nutritional gaps in the diets of older infants and young children. This study evaluated the accuracy of nutrient declarations on labels of 43 commonly available CPCF in three peri‐urban/urban locations: Khsach Kandal district, Cambodia (*n* = 11); Bandung, Indonesia (*n* = 11) and Guédiawaye and Dakar departments, Senegal (*n* = 21). Label values (LV) from product nutrient declarations were compared to analytical values (AV) derived from laboratory nutrient analysis for macronutrients (carbohydrate, protein and total fat), nutrients of public health concern (saturated fat, total sugar and sodium), and micronutrients of interest (calcium, iron and zinc). European Union guidance for nutrition label accuracy was used to set tolerance ranges for each nutrient LV relative to AV. LV were missing for one or more nutrients in 88.4% (*n* = 38) of the CPCF products and no CPCF met EU tolerance thresholds for all nine nutrients assessed. Over half of products with LV for key micronutrients (55.6%, *n* = 10/18) and macronutrients (54.8%, n = 23/42) met tolerances for LV accuracy. Eighty‐five percent (n = 11/13) of products with LV for nutrients of public health concern were determined to be accurate. Nutrient content claims for iron appeared on 19 (44.2%) of the 43 products. Of the products which made an iron content claim, 26.3% had inaccurate LV with the majority of these containing less iron than declared. Regulatory action is needed to ensure that CPCF labelling communicates complete and accurate nutrient content information that enables caregivers to make informed decisions for feeding older infants and young children.

## Key messages

- None of the 43 commercially produced complementary food (CPCF) products assessed met tolerance thresholds for nutrient label value accuracy, either because of missing nutrient label information or discrepancies with laboratory derived analytical values.
- The proportion of accurate nutrient label values for individual nutrients ranged from 54.2% (*n* = 13/24) for calcium to 94.7% (*n* = 36/38) for sodium.
- While infant cereals generally had high proportions of accurate label values for the nine nutrients assessed, fewer than half of snacks/finger foods (e.g. rusks/biscuits) had accurate label values for protein and calcium and only a third were accurate for zinc.
- Nutrient content claims were prevalent across the 43 CPCF products, which can create a ‘health halo' (the perception that something is healthy without direct evidence) for these products. However, over half of products with nutrient content claims for iron and calcium were found to contain lower levels of these micronutrients when compared to analytical values. These products were found to overstate iron content and calcium content by a median of 4.71 mg per 100 g product and 101.7 mg per 100 g product, respectively.
- Nutrient content claims and nutrient declarations should be regulated and monitored by national authorities to ensure accurate labelling of CPCF. Manufacturers of CPCF have a responsibility to provide consumers with correct product information and should be accountable for the content of CPCF nutrient declarations.

## 1 INTRODUCTION

The first 2 years of life represent a complex and essential period for human nutrition. Exclusive breastfeeding is recommended for the first 6 months of life, after which nutrient‐dense complementary foods should be gradually added to a child's diet, with continued breastfeeding for up to 2 years or beyond (World Health Organization, 2003). This transitional complementary feeding period often corresponds with micronutrient deficiencies and growth faltering among young children (Victora et al., 2010), indicating the importance of optimal complementary feeding strategies worldwide.

Because of their small gastric capacity, older infants (6–12 months) and young children (12–36 months) can only eat limited quantities of complementary foods, making the nutritional quality of these first foods a high priority. Adequate intakes of micronutrients, including iron, calcium and zinc, are critical for optimal growth and development (Osendarp et al., 2016). However, complementary feeding diets in low‐ and middle‐income countries (LMIC) show room for improvement. Fewer than half of young children 6–23 months of age in East Asia and the Pacific consume a minimum acceptable diet, indicating adequate quantity and diversity in the diet, while this figure reaches only 9.4% in Western and Central Africa (Gatica‐Domínguez et al., 2021). Market survey research reveals that commercially produced complementary foods (CPCF) have large market share in high‐income countries (Ghisolfi et al., 2013; Theurich, 2018) and these products are becoming increasingly popular in low‐ and middle‐income countries (Abizari et al., 2017; Bassetti et al., 2022; Tuan et al., 2017). CPCF are commonly fed to older infants and young children in urban areas of Indonesia and Senegal. In a study among 6–35 month‐old children in Bandung City, Indonesia, 37.4% consumed a CPCF in the previous day (Green et al., 2019). In Dakar Department, Senegal, half (49.1%) of 6–23 month‐old children consumed a CPCF in the previous day (Feeley et al., 2016). Data gathered from a cross‐sectional study conducted in Phnom Penh, Cambodia in 2013 found that 5.4% of 6–23 month olds consumed CPCF and 29.3% of mothers observed a promotion for CPCF (Pries et al., 2016). While there is a lack of current evidence on CPCF consumption among young children in urban Cambodia, it is likely that consumption of these products has risen over the last decade, given that even peri‐urban areas are seeing an increase in commercial food consumption. A recent study found high consumption of unhealthy commercial food and beverages in peri‐urban Cambodia: 97.4% of young children at 15–19 months of age had consumed such products in the previous week (Hinnouho et al., 2023). Inadequate intakes of calcium among infants and young children in Cambodia has led to recommendations for the use of fortified foods to achieve optimal complementary feeding (Gibbs et al., 2014). While it has been suggested that the use of well‐formulated commercial complementary foods may be an acceptable strategy to meet the required intakes of vitamins and minerals among children in LMIC (Osendarp et al., 2016), it is essential that they are appropriately fortified to meet this goal.

While CPCF can provide a source of critical micronutrients, some CPCF have been found to also contain excessive levels of nutrients of public health concern (identified as saturated fat, total sugar and sodium; World Cancer Research Fund International, 2019). High quantities of both sugar and sodium have been identified in CPCF in Malta and the United States (Elliott & Conlon, 2015; Maalouf et al., 2017; Pace et al., 2020), and added sugars are common in CPCF sold in Europe, contributing approximately one‐third of products' total calories (Hutchinson et al., 2021). Some types of CPCF, such as ‘toddler dinners/meals', have also been observed to contain high levels of saturated fat (Maalouf et al., 2017). These nutrients add to the palatability of CPCF, but have the potential to shape children's later taste preferences, thereby contributing to the risk of child obesity and potentially adulthood cardiovascular diseases (Baker & Baker, 2015). Both the Southeast Asia and the West Africa regions contend with the double burden of malnutrition, where high rates of child undernutrition coexist with increasing rates of obesity and diet‐related non‐communicable diseases (Haddad et al., 2015; Mwangome & Prentice, 2019; Onyango et al., 2019).

Accurate and complete nutrient composition labelling of CPCF is necessary to communicate products' contributions to diets of older infants and young children, both for nutrients essential for growth and development and for nutrients that should be consumed in moderation. Evaluating the accuracy of nutrient declarations on labels is crucial for understanding whether manufacturers are accurately reporting the composition of foods intended for older infants and young children. This information is especially important when products present nutrient content claims on labels, which are commonly found on CPCF (Koo et al., 2018; Menon et al., 2021) and can create a ‘health halo' (the perception that something is healthy without direct evidence) that influences caregivers' product purchase decision‐making (Abrams et al., 2015; McCann et al., 2022). Research investigating the actual nutrient content of CPCF products relative to label nutrient declarations is limited, particularly in LMIC, with even fewer studies interpreting the degree of error in discrepancies between these values and how this can be applied in compliance monitoring of products. In LMIC contexts, where the nutrient‐density of complementary foods can be limited and appropriate fortification therefore crucial, little is known about the accuracy of CPCF nutrient declarations.

This study aimed to assess whether the most commonly available commercial complementary foods in three urban low‐and‐middle income country settings contained key nutrients in the amounts expressed on product labels. The study objectives included: 1) to summarise the nutrient label values and nutrient content claims for macronutrients, key micronutrients, and nutrients of public health concern on labels of CPCF available in Cambodia, Indonesia and Senegal; 2) to determine laboratory derived analytical values for the selected nutrients; and 3) to evaluate the accuracy of label values compared with analytical values for the selected nutrients.

## 2 METHODS

This study involved a cross‐sectional assessment of products available in Khsach Kandal district, Cambodia; Bandung, Indonesia; and Guédiawaye and Dakar departments, Senegal. These geographies were selected to provide several contexts in the Southeast Asia and West Africa regions, as well as peri‐urban and urban sites, where product availability may vary. Products were purchased in June 2020–June 2021 and laboratory analysis conducted in July 2020–July 2021.

### 2.1 Product selection

CPCF were defined as locally manufactured or imported commercially produced foods or beverages specifically marketed as suitable for feeding older infants (6–12 months of age) and young children (1–3 years of age). Products were considered to be CPCF if they met one of the following criteria: 1) recommended for introduction at an age of less than 3 years; 2) labelled with the word's ‘baby’, ‘infant’, ‘toddler’, ‘young child’, or synonym; 3) label with an image of a child who appears to be younger than 3 years of age or who is feeding with a bottle; or 4) in any other way presented (through text or images on the label) as being suitable for children under the age of 3 years (WHO, 2016, 2017).

Products carrying the same brand name but different sub‐brands, descriptive names, age recommendations, age categories, flavours or made by different manufacturers were treated as a unique product. If single‐serving and multi‐serving packages of the same product were available, the single serving package was included. CPCF were excluded from the selection process if a product required refrigeration/had a shelf life shorter than 4 months (to ensure product integrity during shipping), did not provide any label information in a local language (Khmer, Bahasa Indonesia, French and Wolof) or English, or if an insufficient number of packages of the product were available for purchase.

To identify the most commonly available products, CPCF product availability was evaluated across a sample of stores in each location. In Khsach Kandal district, Cambodia, nine out of the district's 18 communes were selected, including a purposive sample of the three most urban communes and a random sample of six of the 15 remaining communes. For each of the communes selected, research assistants visited the village considered to be the most commercially developed (likely to have the most stores) in its commune. Once there, the field workers went to the identified roads and located all stores selling baby goods (e.g., baby stores), medicines (e.g., pharmacies), or commercially produced foods (e.g., grocers, corner/convenience stores and kiosks) along each road within the village boundaries. In Bandung, Indonesia, research assistants visited 33 purposively selected small stores in closest walking distance to public sector health facilities and 10 large retail outlets purposively selected for their large variety of products following an international protocol (WHO & UNICEF, 2017). Large stores were purposively sampled in consultation with local officials and non‐governmental organisations working on child health, and included four grocery stores/supermarkets, four hypermarkets and two baby stores. The 10 locations were chosen for their wide variety and volume of products that would be representative of availability in Bandung. Small stores included corner stores (warung/kiosks), neighbourhood cooperative grocery stores (koperasi), minimarts and pharmacies (apotiks). In Senegal, store scoping was conducted across Dakar and Guédiawaye Departments. In Dakar Department, local researchers compiled a list of larger stores (supermarkets, hypermarkets and pharmacies) that were anticipated to have wide availability of CPCF products. Within this list, independent stores were exhaustively sampled, and for chain stores, the store that stocked the greatest variety of CPCF products was purposively sampled. In peri‐urban Guédiawaye Department, store scoping found that CPCF points‐of‐sale included only one larger store (a supermarket), and four types of smaller stores (superettes, small pharmacies, gas station boutiques and neighbourhood boutiques). Only a small number of superettes and gas station boutiques were identified and exhaustively sampled. Multiple pharmacies and neighbourhood boutiques were identified in each commune. Therefore, the two largest pharmacies and neighbourhood boutiques per commune were purposively sampled. A total of 10 larger stores were visited in Dakar Department and 31 stores (1 larger and 30 smaller) were visited across Guédiawaye Department. In total, 25, 43 and 41 stores in Cambodia, Indonesia, and Senegal, respectively, were identified as selling CPCF and visited for product purchasing.

In each country, the same research assistants visited all stores and purchased all CPCF products available. All unique CPCF available for sale were recorded at each store visited, and prevalence of availability across stores calculated. Based on this product availability assessment in each of the three country locations, the most commonly available shelf‐stable CPCF products were purposively sampled for inclusion in this study across three CPCF product categories: 1) dry or instant cereals/starch, 2) pureed foods/meals, and 3) snacks/finger foods. A total of 43 CPCF products were selected for this study: 21 from Senegal, 11 from Cambodia and 11 from Indonesia.

### 2.2 Data extraction

CPCF product labels were photographed and scanned following previously used procedures (Pereira et al., 2016). All relevant data from the product labels were entered into Microsoft Excel datasheets, including nutrient declarations per 100 g of product as sold for carbohydrates, protein, total fat, saturated fat, total sugar, sodium, calcium, iron and zinc. This data was defined as the ‘label value’ for each nutrient. The presence of nutrient content claims on labels was also recorded. As defined by Codex Alimentarius, these included any representation which states, suggests or implies that a food has particular nutritional properties including, but not limited to, the content of energy, protein, fat, carbohydrates, vitamins and minerals (Codex Alimentarius Commission, 2004). The place of manufacture was also extracted and products were recoded as either ‘manufactured in‐country’ or ‘imported’. Extracted data underwent a 10% error check to ensure accuracy, involving a comparison of extracted data to label scans and photos. Missing nutrient label values were noted.

### 2.3 Laboratory nutrient analysis

To meet the minimum quantity (150 g) required for the laboratory analysis, six packages of each CPCF were purchased. All packages of each CPCF were sent to Eurofins Food Testing Singapore, an accredited laboratory in accordance with the recognised International Standard ISO/IEC 17025:2017 *General requirements for the competence of testing and calibration laboratories*, for nutrient analysis of nine nutrients: carbohydrates, protein, total fat, saturated fat, total sugar, sodium, calcium, iron and zinc. The micronutrients: iron, zinc and calcium were selected for analysis because nutrient density for these three nutrients is commonly a concern among infants and young children's diets in low‐ and middle‐income countries (Dewey, 2013; Gibbs et al., 2014; Osendarp et al., 2016), and ensuring accurate labelling is, therefore, vital for consumers. Industry‐standard methods approved by Association of Official Analytical Chemists (AOAC) International were used to determine nutrient content by weight per 100 g of product (AOAC International, 2019). Total fat, including saturated fat, was analysed by gas chromatography, protein by the Dumas method, and carbohydrate by subtraction. Total sugar was assessed by ion chromatography and minerals (calcium, iron, sodium and zinc) by ICP emissions spectrometry. Nutrient laboratory analysis results were provided in grams (g) of carbohydrate, protein, total fat, saturated fat, sodium, and total sugar per 100 g of product, and milligrams (mg) of calcium, iron and zinc per 100 g of product. This data was defined as the ‘analytical value’ for each nutrient. Products from all three locations were sent to the same laboratory for analysis.

### 2.4 Data analysis

To evaluate the accuracy of label values, the European Commission's Guidance Document for Competent Authorities for the Control of Compliance with EU Legislation on: Regulation No 1169/2011 (European Commission, 2012) was used to determine whether analytical values and label values for each nutrient were in agreement. Though no national or international standards exist for what is considered an acceptable variance in nutrient content for CPCF, this EU guidance was deemed the most comprehensive and specific framework for evaluation of nutrition label accuracy available. A previous study utilised this guidance to compare agreement between label values and analytical values of total sugar in packaged foods (Yusta‐Boyo et al., 2020), demonstrating its utility for the purpose of determining label value accuracy. Rounding rules and tolerance calculations from the EU guidance were used to identify acceptable tolerance ranges for each nutrient label value, taking into account the presence of nutrient content claims, any fortification or added sources of the nutrient in question and the declared grams of nutrient per 100 g of product. Analytical values for each nutrient were compared to this tolerance range; if the analytical value did not fall within this range, the label value was considered to be inaccurate. Analysis was conducted at the nutrient level, but trends in results were also summarised by three groupings of nutrients: 1) macronutrients (including carbohydrate, protein and total fat), 2) nutrients of public health concern (including saturated fat, total sugar and sodium), and 3) micronutrients of interest (including calcium, iron and zinc). Data were analysed and summarised using Stata Statistical Software Release 17 (StataCorp, 2021).

## 3 RESULTS

Label values were missing for one or more nutrients on 88.4% (*n* = 38) of the CPCF labels, with a range of 1–8 nutrients missing out of the nine nutrients assessed in this study. Only one product was missing label values for all three macronutrients (carbohydrate, protein and total fat). Missing label values were more variable for nutrients of public health concern, with 11.6% (*n* = 5) of products missing sodium content but 53.5% (*n* = 23) and 41.9% (*n* = 18) missing saturated fat and total sugar content, respectively. Key micronutrients were the most commonly missing label values; with 44.2% (*n* = 19), 39.5% (*n* = 17) and 55.8% (*n* = 24) of CPCF products missing information on calcium, iron and zinc content, respectively.

Product information, label values and analytical values, as well as agreement between these values, are presented in Table 1. Varying levels of label value accuracy were observed among CPCF across the three countries, particularly for protein, total sugar and key micronutrients. While nearly all CPCF in Indonesia (90.0%, *n* = 9/10) and Senegal (85.7%, *n* = 18/21) met EU nutrient tolerances for protein, all 11 CPCF from Cambodia had inaccurate label values. In Cambodia, all 11 of these inaccurate protein label values were lower than analytical values determined by laboratory testing. Of products that declared label values for total sugar content, half (*n* = 4/8) from Indonesia and 40.0% (*n* = 2/5) from Cambodia had inaccurate label values, with total sugar analytical values higher than label values. Conversely, nearly all (91.7%, *n* = 11/12) CPCF from Senegal that declared total sugar content LV were accurate. All 10 CPCF products in Cambodia which had iron label values were compliant with the EU tolerance thresholds, while all of products observed to have inaccurate label values (14.0% *n* = 6/43) came from Indonesia and Senegal. Of four products in Indonesia that did not have accurate iron label values, two overstated iron content while two understated it. The two CPCF in Senegal with inaccurate iron label values overstated iron content. By contrast, none of the seven products with label values for calcium in Indonesia were accurate, while 88.9% (n = 8/9) and 62.5% (*n* = 5/8) of CPCF had accurate label values for calcium in Cambodia and Senegal, respectively.

**Table 1:** CPCF product information and nutrient declaration label values (LV) and analysed values (AV), by country.^a^, ^b^

Brand/product Name & flavor	Product category	Place of manufacture (in‐country or imported)	Carbohydrate (g/100 g)		Protein (g/100 g)		Total fat (g/100 g)		Saturated fat (g/100 g)		Total sugar(g/100 g)		Sodium (mg/100 g)		Calcium (mg/100 g)		Iron (mg/100 g)		Zinc (mg/100 g)	
			LV	AV	LV	AV	LV	AV	LV	AV	LV	AV	LV	AV	LV	AV	LV	AV	LV	AV
**Senegal (n = 21)**																				
AIA – LTDA Babylac, wheat and milk	Instant cereal	Imported	76.0	80.1	10.4	11.9	4.6	5.0	2.8	2.9	_	37.9	58	66	382	388	13.0	17.3	_	1.1
Nestlé Cerelac, wheat and milk	Instant cereal	Imported	71.0	70.4	14.0	15.1	10.0	9.4	_	1.0	_	25.2	193	215	437	459	8.5	12.4	3.0	3.7
Bledina SAS Bledine, fruit and milk	Instant cereal	Imported	69.1	72.7	13.8	14.4	8.4	8.1	3.9	4.1	33.2	33.5	100	99	697	678	9.0	8.9	4.5	5.6
Cigal Vitaruy, multivitamin and milk	Instant cereal	In‐country	68.0	74.7	16.0	9.7	10.0	11.0	_	4.2	_	35.3	_	229	_	119	16.0	4.4	_	0.8
AIA/SACNPJ Babybom, rice banana apple	Instant cereal	Imported	83.0	87.2	4.1	6.4	0.0	1.6	0.0	0.5	_	17.9	80	12	151	197	9.0	14.3	_	7.4
Nutrimental Nutribom, honey and wheat	Instant cereal	Imported	75.0	86.1	10.0	9.8	0.8	0.9	0.3	0.2	_	31.8	160	205	_	297	15.0	21.0	7.0	9.4
Agro Saafi Saafilac, multicerals	Instant cereal	In‐country	69.3	75.0	16.4	12.4	7.8	4.2	_	1.5	_	27.4	53	224	616	716	14.0	7.3	0.5	1.6
Molinos el Gaunche Forza	Instant cereal	Imported	76.0	83.0	10.5	11.8	2.3	2.0	_	0.4	_	0.7	_	75	38	35	3.4	3.2	2.0	1.9
Senfoods SA Melolac	Instant cereal	In‐country	54.2	71.2	12.0	14.5	7.8	9.5	_	3.4	_	32.6	97	81	298	370	8.8	11.6	4.9	6.8
Agro‐Food Industrie Vita Meal Baby, wheat milk cocoa	Instant cereal	Imported	69.3	78.0	14.4	15.3	1.3	1.4	_	0.5	17.8	29.6	149	130	315	534	18.8	23.4	14.4	16.4
HiPP Biologique Délices du Jardin, vegetable gardener	Pureed food	Imported	4.7	7.4	1.2	1.4	1.3	1.2	0.1	0.1	2.2	2.9	20	34	_	18	_	0.3	_	0.2
Blédina SAS Blédina, fruit cocktail	Pureed food	Imported	13.0	15.2	0.4	0.6	0.2	0.1	0.1	0.0	9.9	11.6	3	2	_	7	_	0.2	_	0.0
Nestlé NaturNes, pumpkin	Pureed food	Imported	7.1	7.3	0.8	0.8	0.7	0.7	0.1	0.0	3.4	2.6	20	19	_	24	_	0.2	_	0.1
Le Lionceau SARL, banana millet	Pureed food	In‐country	15.0	15.3	0.9	1.4	0.2	0.1	_	0.0	11.6	11.6	0	2	_	9	_	0.4	_	0.2
Vicky Food Products Be Plus, fruit with Marie biscuit	Pureed food	Imported	15.0	16.4	0.8	0.9	0.0	0.3	0.0	0.0	12.0	11.6	12	12	_	6	_	0.2	_	0.0
Interdis My Baby Bio, apple mango	Pureed food	Imported	13.0	12.6	0.0	0.5	0.0	0.0	0.0	0.0	11.0	10.4	5	2	_	6	_	0.2	_	0.0
Intelma SARL Babypot, chicken tomato rice	Pureed food	In‐country	7.6	9.2	3.4	3.4	1.3	0.3	_	0.1	_	0.7	_	47	_	15	_	0.5	_	0.2
Blédina SAS Blédina Blédichef, creamy spinach and pacific salmon puree	Pureed food	Imported	7.8	9.1	2.7	2.4	2.2	2.0	0.7	0.6	0.8	1.0	100	77	_	31	_	0.4	_	0.2
HiPP Biologique Les Menus Plaisirs, tomoatoes pasta veal	Pureed food	Imported	7.4	8.3	2.9	3.0	2.6	2.1	0.5	0.4	1.1	1.3	140	134	_	12	_	0.3	_	0.4
Blédina SAS Blédina, with chocolate chips	Snack/finger food	Imported	76.0	76.3	6.1	7.1	13.2	11.9	8.2	7.5	24.7	22.0	190	141	_	31	_	1.3	_	0.5
Le Lionceau SARL Millet, cinnamon	Snack/finger food	In‐country	49.4	60.9	6.2	7.4	23.2	25.9	_	21.1	17.0	15.5	40	15	_	17	_	1.5	_	0.5
**Indonesia (n = 11)**																				
Nestle Cerelac, mung beans	Instant cereal	Imported	68.0	69.4	16.0	16.4	9.0	8.7	_	1.9	24.0	21.2	130	129	270	585	7.5	13.9	3.5	5.8
Nestle Cerelac, bananalicious	Instant cereal	Imported	68.0	71.5	14.0	15.0	9.0	8.5	_	3.4	40.0	39.2	140	128	225	526	9.8	12.6	2.1	3.3
Heinz, apple & mango	Pureed food	Imported	14.3	13.6	0.0	0.7	0.0	0.0	_	0.0	11.4	11.6	14	2	0	5	_	0.2	_	0.0
Heinz, summer fruits gel	Pureed food	Imported	14.3	12.9	0.0	0.5	0.0	0.0	_	0.0	8.6	11.8	29	10	_	26	_	0.3	_	0.1
Heinz, tender beef with vegetable mash	Pureed food	Imported	8.6	7.4	2.9	3.0	0.0	1.2	_	0.6	_	2.4	14	11	_	10	_	0.4	_	0.5
Promina, milky brown rice	Instant cereal	In‐country	70.0	74.5	16.0	14.9	7.0	5.8	_	2.6	18.0	35.4	120	360	495	404	9.8	8.2	5.3	4.8
Promina, free‐range chicken, tomatoes, carrots	Instant cereal	In‐country	80.0	78.7	12.0	11.8	2.4	1.9	0.8	0.9	8.0	7.7	380	900	360	204	12.0	2.4	4.9	2.1
Milna, Kinder pudding, chocolate	Snack/finger food	In‐country	81.8	81.3	9.1	7.8	6.8	6.4	_	2.6	18.2	28.2	409	21	398	296	14.3	7.9	10.9	7.2
Sun Milk Marie Biscuit	Snack/finger food	In‐country	75.0	75.2	8.3	8.5	8.3	11.0	4.2	6.1	16.7	21.2	375	307	563	514	3.4	9.8	_	3.2
Gasol, 100% mung bean, organic flour	Instant cereal	In‐country	130.0	62.8	45.0	24.4	5.0	1.2	_	0.4	_	2.7	_	6220	_	62	_	4.2	_	2.7
Milna Kinder, chocolate	Pureed food	In‐country	_	76.0	_	6.9	_	10.9	2.0	8.7	_	41.2	_	125	_	563	_	2.4	_	0.9
**Cambodia (n = 11)**																				
Milna Bubur Bayi, chicken & corn soup	Instant cereal	Imported	72.5	68.5	12.5	16.2	7.5	8.2	7.5	8.2	12.5	15.3	300	274	438	517	16.6	22.6	6.0	6.1
Milna Bubur Bayi, banana	Instant cereal	Imported	72.5	67.5	12.5	16.9	10.0	9.4	_	3.2	12.5	24.4	225	235	469	543	16.6	17.6	5.6	6.5
Nestle Cerelac, rice and soybeans	Instant cereal	Imported	67.0	65.7	15.0	20.2	9.0	9.0	2.6	2.4	_	22.5	150	142	420	540	10.0	12.1	2.5	4.0
Nestle Cerelac, wheat and honey	Instant cereal	Imported	84.0	79.9	10.0	16.1	1.4	1.2	0.2	0.3	_	28.6	140	126	235	228	15.6	21.1	_	0.7
Nestle Cerelac, wheat with fish and spinach	Instant cereal	Imported	68.6	61.9	16.0	22.7	9.8	10.0	_	4.0	_	23.4	240	309	370	511	10.0	12.5	2.0	3.4
France Lait Diastase	Instant cereal	Imported	69.8	64.8	15.0	21.1	7.0	6.8	_	2.6	_	31.6	188	147	720	667	5.5	4.8	5.5	6.8
Milna Baby Biscuit, orange	Snack/finger food	Imported	77.3	71.8	9.1	15.8	6.8	6.7	_	311.0	22.7	30.3	23	21	1	0	9.2	9.6	9.5	7.7
Gerber Puffs, blueberry	Snack/finger food	Imported	85.7	78.6	0.0	15.6	0.0	1.6	_	0.4	14.3	14.3	0	25	_	24	20.0	25.2	_	2.5
AFC Nutrition Crunchy Crackers, vegetable flavour	Snack/finger food	Imported	65.7	55.6	6.9	20.5	24.3	20.0	11.0	8.5	12.2	10.3	469	473	265	288	_	0.7	_	0.3
Gerber Lil'Crunchies, apple & sweet potato	Snack/finger food	Imported	71.4	51.0	0.0	23.9	28.6	22.8	_	2.0	_	6.4	71	64	_	16	24.3	33.4	_	0.8
Happy Baby Superfood Puffs, purple carrot & blueberry	Snack/finger food	Imported	85.7	83.3	0.0	9.8	0.0	0.7	0.0	0.3	_	4.9	0	79	286	608	12.9	18.0	2.9	5.9

^a^ Inaccurate label values highlighted in orange (LV < AV) and blue (LV > AV).

^b^ Dash (‐) indicates missing label value.

The proportion of products whose label values met the EU tolerance thresholds for each of the nine nutrients are presented in Table 2. No CPCF met EU tolerance thresholds for all nine nutrients assessed. There was variation in accuracy of label values when considering the three categories of nutrients assessed. Just over half (54.8%, *n* = 23/42) of CPCF declaring carbohydrate, protein and total fat content had accurate label values for these macronutrients (Table 2). Two‐thirds (*n* = 4/6) of products with inaccurate total fat labels understated total fat content by a median of 1.7 g per 100 g of product.

**Table 2:** Proportion of commercially produced complementary foods with nutrient label values (LV) meeting EU nutrient tolerance thresholds (*n* = 43).

Nutrient	Products with LV % (*n*)	LV met accuracy tolerance % (*n*)^a^
Carbohydrate	97.7 (42)	83.3 (35)
Protein	97.7 (42)	64.3 (27)
Total fat	97.7 (42)	83.3 (35)
All macronutrients	**97.7 (42)**	**54.8 (23)**
Saturated fat	46.5 (20)	90.0 (18)
Total sugar	58.1 (25)	72.0 (18)
Sodium	88.4 (38)	94.7 (36)
All nutrients of public health concern	**30.2 (13)**	**84.6 (11)**
Calcium	55.8 (24)	54.2 (13)
Iron	60.5 (26)	76.9 (20)
Zinc	44.2 (19)	73.7 (14)
All key micronutrients	**41.9 (18)**	**55.6 (10)**
All nutrients	**11.6 (5)**	**0 (0)**

^a^ Sample sizes for values in this column correspond to the number of products with LV in the previous column.

Approximately half (55.6%, *n* = 10/18) of products with label values for key micronutrients (calcium, iron and zinc) met tolerances for label value accuracy. Nearly half (45.5%, *n* = 5/11) of products with inaccurate calcium labels understated calcium content by a median of 264.0 mg per 100 g of product. Two‐thirds (*n* = 4/6) of products with inaccurate iron labels overstated iron content by a median of 6.6 mg per 100 g of product. Of the five products with inaccurate zinc labels, two overstated zinc content by a median of 3.2 mg per 100 g of product.

A greater proportion of products with label values for nutrients of public health concern (saturated fat, sodium and total sugar) met the EU guidance tolerance thresholds, with 84.6% (*n* = 11/13) of products with label values determined to be accurate. The seven products with inaccurate total sugar label values understated total sugar content by a median of 10.4 g per 100 g of product.

Infant cereals had high proportions of accurate label values for the nine nutrients assessed. Nearly all infant cereals with label values were accurate for carbohydrate (87.5%, *n* = 18/21), total fat (81.0, *n* = 17/21), saturated fat (100.0%, *n* = 8/8), sodium (94.4, *n* = 17/18), iron (80.0% *n* = 16/20) and zinc (81.3% *n* = 13/16), while half of products had accurate label values for protein (52.4%, *n* = 11/21), total sugar (62.5% *n* = 5/8) and calcium (61.1% *n* = 11/18). Pureed food label values were generally accurate for macronutrients and nutrients of public health concern, but could not be determined for key micronutrients due to missing label information. Between 85.7%‐100.0% of purees with label values met EU tolerance thresholds for carbohydrate, protein, total fat, saturated fat, sodium, and total sugar. Only one pureed food provided any label values for key micronutrients; this CPCF, which had a calcium label value, did not meet the EU tolerance threshold. Fewer than half of snacks/finger foods had accurate label values for protein (44.4% n = 4/9), calcium (40.0%, *n* = 2/5) or zinc (33.3%, *n* = 1/3) and only 57.1% (*n* = 4/7) had accurate label values for total sugar. A higher proportion of snack/finger foods (66.7%‐89.9%) had accurate label values for other nutrients, including carbohydrate, total fat, saturated fat, sodium and iron.

Over half of all CPCF products included in the study (53.5%, *n* = 23) made a nutrient content claim. Nutrient content claims were most common for iron, appearing on 19 (44.2%) of the 43 products. Fourteen of the products that made iron content claims (73.7%) met the EU guidance tolerance for accuracy in labelled iron content. Of the products that made an iron content claim and had inaccurate label values, 60.0% (*n* = 3/5) had less iron than expressed in the label value. Calcium was the next most common nutrient content claim, occurring on 25.6% of products (*n* = 11). Six of these products (54.5%) had inaccurate label values which claimed higher calcium content than found in than analytical values. Five CPCF made a zinc content claim, one of which had an inaccurate label value overstating the actual quantity of this mineral in the product. Four products made a protein content claim, three of which had accurate label values. The one product with an inaccurate value was found to have less protein than expressed on the label. One claim each was found for total fat and carbohydrate, both of which had accurate label values. No content claims were observed for saturated fat or total sugar, and the only product with a sodium content claim met the EU tolerance range.

All 21 products from Senegal had label information for macronutrients (carbohydrate, protein, total fat); six of these products were manufactured in‐country and 15 were imported. Of the six local products, 66.7% (*n* = 4/6) had accurate labels for macronutrients. Of the 15 imported products, 86.7% (*n* = 13/15) had accurate labels for macronutrients. Only 12 products (57.1%, *n* = 12/21) from Senegal had label information for all nutrients of public health concern; two of these products were manufactured in‐country and 10 were imported. Of the products that could be assessed, the two products manufactured in‐country had accurate labels and 90% (*n* = 9/10) of the imported products had accurate labels. Seven products (five imported and two in‐country) from Senegal had label information for all key micronutrients (calcium, iron, zinc); the two products manufactured in‐country and three of the five imported products were accurate.

Ten out of 11 products (90.9%) from Indonesia had label information for all macronutrients; five of these products were manufactured in‐country and five were imported. Eighty percent (*n* = 8/10) of the products from Indonesia that could be assessed had accurate labels for macronutrients; three of the five products manufactured in‐country were accurate and all five imported products were accurate. Three products manufactured in‐country (27.3%, *n* = 3/11) had complete label information for all nutrients of public health concern and only one (9.1%, *n* = 1/11) had accurate label information. None of the five products (two imported and three in‐country) with complete label information for key micronutrients met the accuracy tolerance. Only three products (27.3%, *n* = 3/11) from Indonesia had complete label information for all nutrients assessed. All three were manufactured in‐country but none were fully accurate for all nutrients assessed.

All 11 products from Cambodia were imported and 81.8% (*n* = 9/11) had accurate labels for all macronutrients. Of the products that could be assessed for nutrients of public health concern, only three (60.0%, *n* = 3/5) had accurate labels. Seven products had label information for all key micronutrients and six of them had accurate labels. Only five products had complete label information for all nutrients assessed and of these three (60.0%, *n* = 3/5) were fully accurate.

## 4 DISCUSSION

Nutrient declarations are one of the most common components of packaged food labelling worldwide (WHO, 2021), and serves as a key source of information for caregivers of older infants and young children. Accurate label values for nutrients on CPCF products are necessary for caregivers to make informed decisions when purchasing foods for their children, and for policymakers to make sound decisions when determining whether these food products can fill nutritional gaps in local complementary feeding diets. To our knowledge, this is the only study that has evaluated the accuracy of nutrient label values of CPCF available in LMIC contexts using established guidance, with this study among CPCF products in Cambodia, Indonesia and Senegal revealing several areas of concern. While nearly all CPCF assessed in this study provided label values for macronutrients, only half of those label values were accurate. Two‐thirds of products with inaccurate total fat labels understated total fat content by a median of 1.7 g per 100 g of product. Fewer than half of CPCF products provided label values for nutrients of public health concern or for micronutrients critical for older infant and young child development. While 84.6% of CPCF label values were accurate for saturated fat, sugar and salt, only 55.6% of the label values for iron, calcium and zinc were accurate.

Inaccurate or missing label values for total sugar among CPCF were prevalent in this study. Only 58.1% of CPCF products in this study provided total sugar label values, and nearly one‐third of products that did have sugar label values were inaccurate, with analytical values indicating greater total sugar content than what was declared on the package. Alarmingly, all seven products with inaccurate total sugar label values understated sugar content by a median of 10.4 g per 100 g of product. Similar findings have been observed in other literature on foods for older infants and young children. A study of CPCF samples from the United States and UK found that products had 15%–337% more sugar than declared on the label (Crawley & Westland, 2017). Another study found widespread inaccuracies in sugar label values on CPCF, with up to 82% more sugar found in analysed samples (Walker & Goran, 2015). Excessive sugar consumption among infants and young children can pose risks for future health and nutrition. Infants and young children have innate preferences for sweet tastes and need multiple exposures to diverse flavours to help encourage varied taste preferences in later childhood (De Cosmi et al., 2017). Some research suggests that older infants who consume a larger proportion of CPCF with added sugars in early life may be predisposed to greater added sugar intakes as pre‐school and school‐aged children (Foterek et al., 2016). Research has already shown high sugar content of some CPCF (Hutchinson et al., 2021; Maalouf et al., 2017; Pace et al., 2020), and the possibility that these products may in fact contain even higher sugar than is declared on product labels is of great concern for infant and young child diets.

Only half of CPCF in this study provided calcium, iron and zinc label values that were accurate relative to analytical values. Inaccurate labels which were found to have a higher declared label value compared to the analytical value, overstated iron and zinc content by a median of 6.6 mg per 100 g of product and 3.2 mg per 100 g of product, respectively. Quantities of calcium, iron and zinc have been found to be variable and often insufficient among CPCF for sale in Senegal (Dimaria et al., 2018), and a study by Masters et al. (2017) found low correlation between label values and analytical values for micronutrients among locally produced CPCF products available in 22 LMIC settings. Calcium is a key component for skeletal accretion and growth of young children. Complementary feeding diets must provide sufficient calcium to account for the high rate of linear growth from 6 to 23 months of age (Shertukde et al., 2022). Iron deficiency is the most common nutrient deficiency among young children around the globe, as well as a prominent contributor to anaemia worldwide (Sundararajan & Rabe, 2021). Choosing iron‐rich complementary foods is an essential component of ensuring sufficient iron status in children between 6 months and 2 years of age as they transition away from exclusive breastfeeding. Zinc is an essential mineral for the rapid growth and cell proliferation which takes place in infancy and early childhood. Older infants and young children are reliant on zinc‐rich complementary foods to meet their needs (Daniels et al., 2018) since breastmilk zinc concentration declines as an infant ages (Pan American Health Organization; WHO, 2003). Higher zinc intake has been found to be protective against underweight and wasting in the complementary feeding period in LMIC contexts (Maciel et al., 2021). Calcium, iron and zinc intakes were also observed to be lower among underweight, wasted, and stunted children at 12 months of age (Maciel et al., 2021). These three nutrients are a priority in the diets of older infants and young children in Cambodia, Indonesia and Senegal, contexts where undernutrition is prevalent and where nutrient density of general diets is limited, and label value accuracy for these key micronutrients in these contexts is therefore critical.

Prior research has highlighted CPCF as a means to improve nutrient adequacy among older infants and young children in Cambodia and Indonesia. Inadequacy of zinc, iron and calcium in diets of older infant and young children in Cambodia has led to recommendations for the use of fortified foods to achieve optimal complementary feeding (Gibbs et al., 2014). Locally available complementary foods in Indonesia have also been found to lack sufficient zinc, calcium and iron (Egayanti et al., 2018; Fahmida et al., 2014), making fortified foods a solution to improve dietary adequacy (Fahmida et al., 2014), particularly in households of lower socioeconomic status (Fahmida & Santika, 2016). While no proposals were found in the literature for use of fortified CPCF as a programmatic solution to address nutrient density of complementary foods in Senegal, some research suggests that Senegalese infants and young children from food insecure households may not be eating sufficient iron‐rich foods as part of traditional complementary feeding diets (Akpaki et al., 2021). Inaccurate nutrient labelling of CPCF has implications for public health research that relies on such values for assessing dietary adequacy among infant and young children who consume these products, with modelling of diets including these products potentially over‐ or underestimating their impact on nutrient intakes. Ensuring that CPCF provide accurate label nutrient values is necessary to ensure their appropriateness and reliability in programme and policy recommendations for complementary feeding.

This study found missing nutrient content information on a majority of CPCF across all three countries. Complete and accurate nutrient declarations on CPCF are necessary for consumers and public health professionals to evaluate the nutritional quality of these foods. Individual caregivers need this information to determine if a CPCF is an appropriate addition to the diets of their older infants or young children. Those tasked with providing recommendations for complementary feeding need to know how a CPCF might contribute to intakes of nutrients which may be lacking in local food sources. Country governments should take this into account by setting and enforcing standards for required nutrient declarations on CPCF products: both manufactured in‐country and imported. National regulations in Indonesia require that quantities of macronutrients (including carbohydrates, total fat and protein), saturated fat, total sugar and sodium be provided in the nutrient declaration of packaged foods (Regulation of Drug and Food Control Agency, 2019). Vitamin and mineral content can be listed if present in an amount at least 2% of the Indonesian recommended dietary allowance per serving. Results from this study indicate that these regulations need to be better enforced, since none of the 11 CPCF purchased from Indonesia provided nutrient label values for all nine of the assessed nutrients. The only three products purchased in Indonesia with complete label information for saturated fat, total sugar and sodium were all manufactured in‐country, however, only one product had accurate label information. Cambodia's *Law on the Management of Quality and Safety of Products and Services No. 126 CL, 26 June 2000* requires food labels to provide information on product composition in Khmer language, but there are no specific mandatory provisions for what nutrients must be declared (Kingdom of Cambodia, 2000). Opportunities to update this law should be sought to ensure comprehensive CPCF nutrient labelling in Cambodia. Senegal does not have legal requirements for nutrient declarations and do not monitor the nutrient content labelling of packaged foods including CPCF. However, a national standard for infant flours adopted in October 2020 does mandate labelling of the nutritional value of energy, protein, lipids, minerals and vitamins (Senegalese Association for Standardization [(ASN], 2020). This standard applies to powdered mixes of tubers, starchy fruits, legumes, or cereal grain designed to be cooked into a porridge and used as a complementary food‐ falling under the category of infant cereals in this study. This standard could be used as a model for more comprehensive requirements for nutrient declarations on all categories of CPCF in Senegal. Our findings indicate that nearly half of the products purchased in Senegal did not have complete labelling information for saturated fat, total sugar, sodium. Of the products that could be assessed for these nutrients of public health concern, two products manufactured in Senegal had accurate labels and 9 out of 10 of the imported products had accurate labels.

Nutrient content claims appeared on over half of the CPCF in this study. The majority of these claims were for key micronutrients, including iron and calcium. Approximately one‐quarter of products with iron (26.3% *n* = 5/19) and calcium (27.2%, *n* = 3/11) claims had inaccurate label values. A recent study of Taiwanese CPCF also found a high prevalence of calcium and iron nutrient content claims and determined no significant difference in iron content between products with an iron nutrient content claim and those without a claim (Koo et al., 2018). This highlights the potential for nutrient content claims to mislead caregivers about the actual content of key micronutrients in CPCF. Recent research has also demonstrated that nutrient content claims influence the perceived healthiness of a product and affect consumer purchase intentions for infant and young child feeding. In a survey of Australian caregivers of children 1–3 years of age, nutrient content claims were the most important contributors to the perceived healthiness of ‘toddler’ snack foods and ultra‐processed ‘toddler milks’ (McCann et al., 2022). In one United States study, researchers found that juice drinks with vitamin C content claims purchased by households with young children were actually more likely to contain higher levels of sugar and calories than juice drinks without these claims (Duffy et al., 2021). Another survey found that vitamin‐fortified snack products with nutrient content claims were perceived as healthier than comparable foods despite having an overall unhealthy nutrient profile (Verrill et al., 2016). Front of pack promotional tactics, such as nutrient content claims, create a ‘health halo’ effect that can be more persuasive to consumers than back of pack nutrient declarations. Evidence from Uruguay found that low‐income mothers reported that they relied on nutrition claims on the front of product labels to determine the healthfulness of foods for their children, finding nutrient declarations on the back of pack to be too complex to interpret (Machín et al., 2016). In semi‐structured focus groups among Southeast Asian mothers, researchers found that trust in government institutions and large multinational brands was related to perceived credibility of iron and calcium health claims on milk powders for children (Tan et al., 2016). Given nutrient content claims influence purchasing behaviours, caregivers of young children may be opting for products with claims that are not appropriately formulated or contain lower/higher nutrient content levels than declared. Nutrient content claims should be regulated by competent national authorities to ensure that they correctly reflect the nutritional features of CPCF.

In lieu of CPCF‐specific guidance, this study utilised the European Commission's Guidance Document for Competent Authorities for the Control of Compliance With EU Legislation on: Regulation (EU) No 1169/2011 (European Commission, 2012) to evaluate the accuracy of nutrient label values declared on products. Products intended for older infants and young children generally do not have specific regulatory schemes against which nutrient label value accuracy can be evaluated. Even in countries where composition, labelling and marketing of products for older infants and young children are explicitly included in regulations, limited guidance is provided on how accuracy of nutrient label values might fit into compliance monitoring (Dreyfuss et al., 2019, Sweet et al., 2016). Other studies evaluating the use of the EU guidance have concluded that it is a useful tool for identifying products which may be contributing to the consumption of nutrients of public health concern, particularly sugar, in amounts beyond what is expressed in nutrient declarations (Yusta‐Boyo et al., 2020).

This study has several limitations. First, due to resources available for this study, only sub‐samples of all CPCF available in each of the three countries were included in this study; covering only 43 products, but a much wider range of products exist on the markets in these locations. These results are not generalisable to all products in each country or beyond the settings where these products were identified. Additional studies on the accuracy of CPCF nutrient label values are needed. Second, this study focused on evaluating the accuracy of label values and not the appropriateness of these label values, or analytical values, for older infant and young child nutritional requirements. This was outside of the scope of the current study but would be a valuable further addition to the discussion of appropriateness of CPCF for complementary feeding in these contexts. Further research is needed to determine if the nutritional composition of CPCF is adequate for this age group in LMIC settings.

## 5 CONCLUSION

This study found that nutrient declarations on the labels of CPCF sold in Cambodia, Indonesia and Senegal often lack complete information on the content of macronutrients, nutrients of public health concern and/or key micronutrients. Of the products which did provide sufficient nutrient label values, none were determined to be comprehensively accurate for the content of carbohydrates, protein, total fat, saturated fat, sugar, sodium, calcium, iron and zinc. Nutrient content claims were present on over half of CPCF products assessed, even though they frequently had inaccurate label values for the claimed nutrients. Cambodia, Indonesia and Senegal are all contexts where CPCF have the potential to improve dietary adequacy among infants and young children, but this requires CPCF nutrient declarations to be complete and accurate. National governments should be encouraged to set standards and to monitor and enforce rules around accuracy of product nutrition labelling. Donors and international organisations in the child nutrition space should be prepared to contribute to filling these gaps where national capacity is limited. Attention should also be paid to the responsibilities held by corporate manufacturers of CPCF to accurately label their products and ensure that nutrient declarations best reflect their actual content.

## AUTHOR CONTRIBUTIONS

Alissa M. Pries conceptualised and oversaw the study. Jane Badham and Anzélle Mulder developed the study protocols, with input from Alissa M. Pries, Katelyn Yuen‐Esco, Eva Juniza, Ndeye Yaga Sy, and Rosenette Kane. Jane Badham and Anzélle Mulder led data collection activities. Eva Juniza, Ndeye Yaga Sy, and Rosenette Kane conducted data collection in Indonesia and Senegal. Katelyn Yuen‐Esco conducted data management and analysis. Mary Champeny drafted the manuscript. All authors reviewed and provided comment on the manuscript before finalisation.

## CONFLICT OF INTEREST STATEMENT

Authors have no conflicts of interest to declare.

## ETHICAL STATEMENT

Not applicable, as this study utilised product label information and no human subjects were involved.

## Data Availability

The data that support the findings of this study are available from the corresponding author upon reasonable request.
